# Overweight and obesity among children in rural areas: The importance of culture

**DOI:** 10.1177/14034948211037877

**Published:** 2021-08-21

**Authors:** Reidun Heggem, Alexander Zahl-Thanem

**Affiliations:** 1 NTNU – Norwegian University of Science and Technology, Norway; 2 Ruralis – Institute for Rural and Regional Research, Norway

**Keywords:** Obesity, overweight, cultural aspects, rurality, Pierre Bourdieu, cultural capital, habitus, social field

## Abstract

**Aims:**

This paper focuses on how social inequality is associated with overweight and obesity in children. There is a lack of research with a focus on an important distinction in social inequality, namely geography. The aim of this study was to reduce this knowledge gap by looking closely at the links between rurality and overweight.

**Methods:**

The findings in this paper are based on in-depth interviews with school nurses and teachers in rural Norway. The focus was on their experiences with and knowledge about overweight and obesity numbers in rural versus urban areas.

**Results:**

We used Bourdieu’s terminology to address the challenges related to urban–rural differences, and found that cultural factors connected to tradition, identity and courtesy play an important role in the rural overweight and obesity discourse.

**Conclusions:**

**Actors and ‘experts’ working with overweight and obesity and national guidelines need to understand rural contexts and customs and address problems of the countryside on rural, not exclusively urban, premises. Different contexts imply different needs when it comes to reducing the inequalities between rural and urban areas regarding overweight and obesity.**

## Introduction

Childhood obesity is among the most serious public health challenges of the 21st century [1], and the impact of overweight and obesity on a variety of non-communicable diseases has been well documented [2]. There is emerging evidence of spatial inequalities in overweight and obesity, especially along a rural–urban categorisation of space. Several studies show that rural residency increases the risk of overweight and obesity in both adolescents and adults [3–5], as well as in children [6–8]. A number of contextual (i.e. characteristics of a place) and compositional (i.e. characteristics of residents) factors have been put forward as potential explanation for these differences between rural and urban areas, with socio-economic inequalities being the most important [9,10]. Few studies have, however, examined how rural culture relates to overweight and obesity among rural children [11]. Focusing on culture is important, not only to identify contributing drivers for rural obesity in children, but also to understand fully how one can implement measures that do not defy rural-specific cultural premises.

For this study, we sought to disentangle some of these issues by interviewing school nurses and teachers working with children in primary schools in rural areas in Norway. These professionals are key actors who observe and interact with the same children over a long period of time, and who thus develop unique knowledge of factors that influence children’s health. For instance, school nurses collect data on children’s weight and have a responsibility to follow up on the children who deviate from desired values, while teachers interact with the children almost every day of the week. How do they perceive the situation of overweight and obesity among children on an urban–rural dimension? In this article, we apply Pierre Bourdieu’s concepts of capital, habitus and social field as a theoretical framework to understand actors’ perceptions of rural overweight and whether these perceptions are related to different social representations of the rural.

### Theoretical perspectives

Pierre Bourdieu’s concepts of habitus, cultural capital and social field refer to how individuals, through local cultural socialisation, possess different basic dispositions. Habitus is defined as a system of dispositions that monitor one’s actions. These dispositions are subconscious, not subject to reflection, and underpin our thinking and doing [12]. Capital represents the values on which social hierarchy is formed. Cultural capital can be defined as a form of knowledge that controls the empathy of agents and their valuation of different cultural expressions [12,13]. Our tastes – what we prefer – can be understood as embodiments of cultures. What we like to eat, what we care about and how much we invest in health will thus depend on our habitus and the type and amount of cultural capital [12]. Bourdieu’s field concept is about the social environment or the social context in which habitus is shaped. Different fields require different skills, dispositions and resources from an actor. Actors are equipped with a habitus that requires knowledge and recognition of the field’s inherent rules [12].

Different practices, including dietary habits, are not entirely freely produced, but rather are subconsciously constructed on the basis of the values and rules – the social representations – that operate in people’s lives [14]. Regarding social representations, two extremes dominate the discourse of rurality and rural childhood [14–16]. First, the idea of the rural idyll reflects an understanding of the rural as better and safer for families, greater freedom for children, supportive communities and being closer to nature [16,17]. There is also the notion of the rural dull or rural horror [14–16]. Here, rural life is defined as less progressive than life in urban societies, as traditional rather than modern and backward looking rather than focused on the future [14]. Rural communities tend to be perceived by young people as traditional, underdeveloped, backward and old-fashioned [18], and urban youth even tend to find the rural primitive [19].

Such representations are often normatively charged [14] and influence both political and individual choices through the notion of habitus. It is essential to study to what extent the actors’ conceptualisations of the rural might be understood as being related to different representations in connection with habitus, capital and social field.

## Methods

Few medical issues can be sufficiently understood through quantitative research alone [20]. Qualitative research methods allow for the exploration of social events and how and why human beings act as they do in their natural context. Qualitative inquiries are increasingly accepted as contributing to a broader understanding of medical science [21,22].

Based on this, we conducted a qualitative study where data were obtained through semi-structured interviews and focus group interviews with key informants (i.e. teachers and school nurses) who work with children with overweight and obesity. The interviews took place in five different rural municipalities in Mid-Norway. The municipalities were chosen with the main objective to ensure a somewhat geographical spread and variation in the level of rurality. The rural areas are remote with variation in population density, centrality, location (i.e. on the coast or inland) and proportion of people employed in tourism and agriculture. School nurses were contacted and recruited through the municipalities’ administration website, while interviews with teachers were organised in collaboration with the municipality’s school administration.

We carried out both individual interviews (*n*=13) and two focus group interviews (*n*=8), resulting in a total of 21 informants interviewed (this included 16 health nurses and five teachers). All of the nurses and teachers were female, and they ranged from their early 20s to late 60s. We stopped interviewing when we approached theoretical saturation, that is, ‘a point where the researchers find nothing new from their collected data from the respondents’ [23]. The interviews were audio recorded and transcribed. All respondents consented to the use of a tape recorder, and the recording was not perceived to have impacted the interviews. All interviewees are given fictitious names in this paper. The interviews were conducted at the informants’ workplace or other communal area and lasted for about 1.5–2 hours.

The analysis process we applied was inspired by the work of Tjora [24] and the stepwise deductive inductive (SDI) strategy that aims at developing codes, categories and concepts as a basis for reaching new understandings of the topic in question. The transcribed interviews were analysed in two steps. In the first step, an overall process of coding based on the themes in the interview guide was conducted. Themes identified were food habits, activity level and body ideal connected to traditions and culture. In this article, we decided to focus on different aspects of food habits, partly because of the importance of food intake when dealing with overweight and obesity [25], but also because of the connections the health nurses made between food habits, rural traditions and obesity/overweight among children. The next level of analysis contained a more thorough coding of the parts of the interviews that referred to food habits. The coding and the grouping of codes [24] led to a closer focus on three themes: the availability of food, the amount of food and the content of the food.

## Results

Almost all informants interviewed in this study described that they felt that people nowadays (including children) use food and snacks to treat themselves with ‘something special’. The informants perceive this trend as both related to food availability and the fact that most people can afford it. The informants argued that this might have consequences for the energy balance equation and, if exaggerated, lead to overweight and obesity. This perceived trend in food habits was argued to be applicable to both rural and urban areas. However, the informants in this study perceived some key differences between rural and urban areas regarding how food is embedded in people’s culture and traditions. These key issues relate to a rural courtesy and a tradition of (a) always having food for visitors, (b) always having enough food and (c) having home-made food with ‘quality’ products (meaning full-fat products). In the following sections, we will elaborate on these issues more thoroughly.

The informants working in rural municipalities stressed the rural importance of serving food in different social contexts.

It’s both for joy and for politeness, to have something to serve. Because I do not believe that the fridges in the cities are filled with cake, but here in the countryside, I think it is both cake and donuts. Ready to be served. (Teacher Rural 1)

There is always a social occasion in the countryside, for serving food. (Health nurses, focus group Rural 1)

The informants stressed that the availability of food to potential guests is part of a ‘rural-specific’ courtesy, which is a continuation of an old tradition of hospitality and generosity in rural areas [26]. Having food available for potential visitors (i.e. friends, family or neighbours) is a natural and necessary part of social interactions. According to the informants, this often involves calorie-rich food, such as a home-made cake, waffles and donuts.

Another perspective on the customs of bringing food to social events is connected to a feeling of community. The quotation below is an example taken from the interview with a teacher working in a rural municipality:

You notice it when you attend a wedding for example. If you are in the countryside, there will be 40 cakes, not because you need 40 cakes, but because everybody brings a cake. (Teacher Rural 2)

The informants describe that during a wedding, not only is the amount of food related to the number of guests, but there is also the custom that everyone brings *something*. The informants connect food traditions to participation in a culture that focuses on the community and unity, togetherness. The concept of *talkot* or ‘dugnad’ (meaning offering a helping hand) is often connected to the countryside as a means for maintaining a shared identity and sense of belonging in a time of increased centralisation [27].

Additionally, embedded in the courtesy was also having enough food. This includes never running out of food, or the idea that no one should ‘leave hungry’. As one informant exemplified, using a wedding as an example:

People in our village enjoy talking about for example weddings in the big cities: ‘The cakes didn’t show up until midnight and there was only *one* cake!’. It’s fun to talk about. They enjoy comparing this to the rural cake table, and that implies ‘City people don’t get it, they do not know how it is done’. (Health nurse Rural 1)

‘. . .one is afraid of running out of cake’. (Teacher Rural 1)

The possible negative effects of the focus on the amount of food, and the need to have enough food, is reinforced by the fact that access to calorie-rich food is easy.

‘In fact, the food tradition actually deteriorated with access to energy-rich food’. (Health nurses, focus group Rural 1).

The consumption of ultra-processed food is rising, and the negative connection between consuming such food and overweight is well established [28]. Easy access to ultra-processed foods is also recognised by the informants in this study. However, rural health nurses experience an additional negative effect when such foods are combined with rural tradition of always serving enough food.

A third distinction that was drawn up was that between home-made and purchased food.

‘When I visit someone for coffee in the countryside, I often get served something home-baked, wheat buns, etc. While visiting friends in the city, they serve dark chocolate or something more modern to accompany coffee. The dark chocolate is healthier, and you eat only a small bit instead of several waffles. It’s more tempting with waffles, but more sophisticated with dark chocolate’. (Health nurse Rural 2)

‘You shall not cheat, you need good butter not light products, because that is not good enough when you bake for celebrations like Christmas’. (Health nurse Rural 3)

The relevance of highlighting the distinction between home-made and purchased cake lies in the idea that the effort made, time spent and being competent as a baker are important. The effort you devote to baking for sharing with others is part of an identity that is valued.

‘It’s about something inherited; you have to do it that way to be considered a good housewife’. (Teacher Rural 2)

In summary, this indicates that there are traditions, customs and courtesies in rural areas that relate to food. These are about serving enough food and real food, meaning home-made with ‘good’ ingredients, both when you invite people and when you are invited to a social gathering. This is presented as important for the cohesion of the community and a potential social loss should it be stopped to overcome obesity.

‘We might lose our soul. It should be an abounding cake-table when you have an infant baptism or confirmation and so on. But you have to distinguish between every day and celebration. That distinction has been erased’. (Health nurse Rural 4)

While these perceived rural-specific values and traditions strengthen a community’s cohesion, it might become a problem if it leads to having energy-rich intake often, when a special occasion suddenly lasts the whole week through. This is an interesting and important distinction. The custom of having an abundance of cake on special occasions is not the problem, rather the problem is that people treat themselves with ‘something special’ all the time. So, according to the informants, there is a need to structure traditions and to set limits better, separating everyday life from celebrations.

It might seem like a paradox that traditions that have been present for a long time suddenly become a possible reason for overweight. The informants explain this apparently paradox with the change in lifestyle:

‘We now have a lifestyle that allow the genes you have predisposed to be expressed. They develop to something else . . . you live differently from what you used to, but you still have a village kind of lifestyle . . . more cake is joining the coffee than in the cities’. (Health nurse Rural 1)

With these changes in lifestyle, connected to easy access to ultra-processed foods and rural traditions as always having enough food for visitors, the total energy intake, according to the informants, exceeds energy output and thus serves as a potential rural and cultural contributing driver of overweight and obesity. The informants emphasise though that social gatherings with accompanying food are essential for the rural identity and that despite its potential negative health impact, the community becomes poorer if we remove traditions and culture.

The informants emphasised the importance of acknowledging and respecting the traditions and customs of rural contexts when working with the overweight and obese. However, they often experienced the opposite. In situations where health problems were discussed, the countryside and the rural areas were often referred to as something uniformly negative:

‘It’s important that one really enter the rural context, and not bring only biased opinions’. (Health nurse Rural 1)

‘It can’t be that cities set the standard for what happens in the rural’. (Health nurse Rural 4)

The informants experienced that public health and ‘overweight issues’ were commonly discussed by health workers and research environments on urban premises. In other words, the informants emphasised the importance of actors working with these issues to make sure that they also discuss the problems of the countryside on rural premises.

## Discussion and conclusions

The premise for this paper is the fact that childhood overweight and obesity are unevenly geographically distributed, and that the challenges are more extensive in rural areas than in urban areas. This implies that we include ‘place’ as a factor when working with health inequality, and it makes visible the need to address rural-specific contextual and compositional factors in order to tackle rural overweight and obesity. This article provides insight into the thoughts and experiences of actors who, through their occupational role as health nurses and teachers, interact and observe children over time in their everyday life. The results show that in addition to the known risk factors, such as the access to energy-rich and ultra-processed foods [28], their focus was the meaning of local, rural custom and tradition as a potentially reinforcing factor for overweight and obesity.

Bourdieu’s concept of habitus is related to culture and identity construction. People are being socialised into a culture that creates an identity, a habitus, which guides them in how they live their lives. Habitus is made up and shaped by the social field around the actor. Different social fields require different kinds of competencies, knowledge and resources of actors. During the interviews, food and food traditions were identified as important markers of the rural social field and the rural identity. The ‘norms’ in this social field were connected both to the setting, the amount and the content of food. For instance, not serving cake in a social gathering or not bringing cake to a social gathering might be ‘breaking the social norms’. In addition, food should preferably be home-made, and one should never run out. This was considered an important cultural capital as a part of the rural habitus where competence and effort to baking yields positive feedback and is appreciated and admired. We see here the contours of a representation of the rural as a ‘rural idyll’ as a supportive community [19].

Some of these food traditions might, according to the informants, have negative implications on people’s health and weight, such as the acceptance of treating yourself with something special and filling the freezer with home-baked cakes in case of unexpected visitors. On the other hand, these are activities that the informants emphasise as having a unifying effect for the community; they create a sense of togetherness, and informants are afraid people are going to ‘lose their souls’ if interventions in work with obesity and overweight interfere with these customs. The rural idyll would crack.

Actors working in public health should be aware of the potential dilemmas that social field, cultural capital and rural habitus represent. Researchers, health authorities and other actors must strive for knowledge about the social norms in the social field they are working in and search for solutions that will not ruin what are considered valuable elements in a community. The informants in our study expressed that this is not necessarily the case. On the contrary, they experience that the focus on work with the challenges of overweight and obesity is influenced by the social representation of the rural dull. Dietary recommendations, national guidelines and interventions implemented by health authorities and others that are unfamiliar or even ignore such traditions, customs and social norms may be perceived as foreign, urban or even hostile and seen as an attack on their values and lifestyles. This might create a basis for a counter-culture against health authorities and other official expert knowledge and make it difficult to get through with knowledge and research.

The informants do not conceptualise the rural as exclusively idyllic or dull. They describe a situation where the two representations collide as the informants describe them as existing side by side. Rural idyll is the representation that is used when they describe the rural community as it is in their eyes. Rural dull is used to describe the rural community as looked on from the outside with urban eyes. A rural habitus is made up of knowledge of both representations, and they are examples of different fields with different rules connected to what is valuable and important. Values and traditions connected to generosity when it comes to food in different aspects are confronted with rules made up in another social field, focusing on experts bound by national guidelines, which rural actors consider urban in its expression. Both representations have to be taken into consideration when working with these topics. One must understand that different contexts imply different needs, and this is the case for the inequalities between rural and urban areas regarding overweight and obesity.
